# Analysis of Allergen-Specific T Cell and IgE Reactivity to Different Preparations of Cow’s Milk-Containing Food Extracts

**DOI:** 10.3390/cells8070667

**Published:** 2019-07-02

**Authors:** Meng Chen, Aaron Sutherland, Giovanni Birrueta, Susan Laubach, Stephanie Leonard, Bjoern Peters, Véronique Schulten

**Affiliations:** 1 Division of Pediatric Allergy & Immunology, Rady Children’s Hospital San Diego, University of California, San Diego, CA 92123, USA; 2 Department of Medicine, University of California San Diego, La Jolla, CA 92093, USA; 3 La Jolla Institute for Immunology, La Jolla, CA 92037, USA

**Keywords:** cow’s milk allergy, baked milk, extensively-heated milk, allergen extract, T cells, IgE

## Abstract

Background: cow’s milk allergy (CM) is among the most common food allergies in young children and is often outgrown by adulthood. Prior to developing a tolerance to CM, a majority of CM-allergic children may tolerate extensively-heated CM. This study aims to characterize the IgE- and T cell-reactivity to unheated CM and the progressively more heated CM-containing foods. Methods: CM-containing food extracts from muffin, baked cheese, custard and raw, pasteurized CM commercial extract were tested for skin prick test reactivity, IgE binding and T cell reactivity as assessed by IL-5 and IFNγ production. Results: the skin prick test (SPT) reactivity was significantly decreased to muffin extract compared to raw, pasteurized CM. Both IgE- and T-cell reactivity were readily detectable against food extracts from all forms of CM. Western blot analysis of IgE reactivity revealed variability between extracts that was protein-specific. T cell-reactivity was detected against all four extracts with no significant difference in IL-5 or IFNγ production between them. Conclusion: our data indicate that despite reduced clinical reactivity, extracts from heated CM-containing foods retain immunogenicity when tested in vitro, particularly at the T cell level.

## 1. Introduction

Cow’s milk (CM) allergy is one of the most common pediatric food allergies, affecting about 2% of young children in the United States [1,2], and its prevalence continues to increase [3]. Although in most cases, CM allergy is outgrown, it still poses a great health risk as avoidance of all CM-containing products is difficult and accidental ingestion is common [4]. Allergic reactions to CM can be very severe, and are estimated to be responsible for up to 13% of fatal food-induced anaphylaxis [5]. Tolerance to CM had previously thought to occur by school age, however, studies from tertiary centers show that a majority may not develop tolerance until adolescence [6,7].

Clinical diagnosis of CM allergy is typically made by a combination of history, diagnostic testing, including skin prick testing (SPT) and food-specific IgE (sIgE) levels, and, if needed, oral food challenges (OFC). Prior to the developing tolerance to unheated CM, one prospective study reported that 75% of CM-allergic children were able to tolerate extensively-heated (baked) CM [8]. Inclusion of baked forms of CM in the diet appears to accelerate the development of tolerance to unheated CM [9]. As a result, encouraging ingestion of heated forms of CM after documentation of tolerance (via OFC or report of regularly tolerating such items at home) has become common practice in the clinical management of CM allergy [10].

The natural course of CM allergy and development of tolerance to baked and then raw forms, (where raw refers to unheated, not unpasteurized) of CM has been clinically well described. However, the mechanism by which heating CM affects allergic reactivity on an immunological level is not fully understood [7]. Heating may denature conformational CM IgE epitopes of heat-labile whey proteins (α-lactalbumin, β-lactoglobulin, and lactoferrin), while not affecting heat-stable proteins, such as casein and bovine serum albumin [11]. Conversely, heating may strengthen some protein bonds, making them more allergenic, or even creating new epitopes. One such process, termed the Maillard reaction, involves enzymatic browning that occurs when roasting peanuts. Another potential mechanism involves the interaction of the protein with the food matrix. Whey proteins can form disulfide bonds with other proteins in the food matrix, potentially blocking IgE binding [12]. Identifying which patients may react to baked CM continues to be evaluated, however a review of studies suggest that very high CM-specific IgE levels or high casein-specific IgE levels may be predictive [13]. So far, studies have not identified CM- or casein-specific SPT to be reliable in predicting baked CM reactivity. The impact of heating and food matrix interaction on allergic T cell responses is yet to be investigated.

T cells are known to play a key role in mediating food allergy [14,15]. While Th2 cells are key players in the pathology of food allergy [16], regulatory T cells (Tregs) are believed to be involved in tolerance induction [17]. In contrast to IgE antibodies, which recognize both linear and conformational epitopes, CD4+ T cells recognize epitopes composed of short (~15 amino acid) peptide sequences presented to them by antigen presenting cells in the context of major histocompatibility complex (MHC) class II presentation [18]. Therefore, CD4+ T cells do not rely on the allergen to be presented in an intact form, and it is unclear how allergen destruction by means of heating may affect T cell reactivity. A study on the T-cell reactivity against birch pollen-related food such as apple, celery and carrot, has reported that cooking these foods did largely abolish IgE binding, however, it did not affect T cell reactivity [19]. Whether the same observations will be made in the case of baked or cooked forms of CM has not been determined.

Understanding the immunological mechanisms of how heated CM-containing foods versus raw, pasteurized CM extracts are recognized by IgE antibodies and T cells will help us to appreciate why the ingestion of baked CM is associated with accelerated onset of tolerance. In this study, we characterized CM-specific IgE- and T cell- reactivity from CM-allergic patients in response to a raw, pasteurized CM extract and progressively more heated CM-containing food extracts (custard, baked cheese and muffin).

## 2. Methods

### 2.1. Subject Population

CM-allergic children ≥6 months of age were recruited from a tertiary care center. Patients provided oral assent if age-appropriate, and their parent or guardian provided written consent. CM allergy was determined by a history of an IgE-mediated allergic reaction to CM and CM-specific SPT wheal ≥3 mm greater than the negative control or CM-specific IgE >0.1 kU/L; or if no history of CM ingestion, by testing suggestive of >95% likelihood of clinical allergy: CM-specific SPT wheal of ≥6 mm in children ≤2 years of age and ≥8 mm in children > 2 years of age, or CM-sIgE ≥ 5 kU/L in children ≤2 years of age and ≥15 kU/L in children >2 years of age [20]. Baseline SPT were performed using commercial CM extract (milk extract from processed, pasteurized cow’s milk) obtained from Greer Laboratories (Lenoir, NC, USA) and fresh CM-containing food extracts as described below. CM-specific IgE levels were measured using ImmunoCAP^®^ (Thermo Fisher Scientific/Phadia, Uppsala, Sweden). For part of the analysis, non CM-allergic controls were recruited from the same center. CM tolerance was determined by parental report of regular unheated CM ingestion without clinical symptoms. The study protocol was approved by the Institutional Review Board at the University of California, San Diego, CA, #121660 and #161563. Patient characteristics are summarized in Table 1. Individual CM-specific and casein-specific IgE and IgG4 titers, extract skin prick test (SPT) results and IgE-SPT correlations are shown in Table 2.

### 2.2. Extract Production and Protein Concentration Determination

The same CM extract used for SPT was used for in vitro studies. CM-containing foods for the extracts were prepared using standardized recipes or purchased (frozen cheese pizza). The milk-containing foods were prepared using commercially available fat-free cow’s milk. For extract preparation, foods were processed as follows: a serving of the cooked or baked food was mixed with ice cold 100 mL Phosphate Buffered Saline (PBS), pH 7.4, with cOmplete™ protease inhibitor cocktail tablets (added as per manufacturer’s guidelines) (Roche Diagnostics, Basel, Switzerland) and blended on ice using a food processor to a smooth emulsion. For the baked cheese extract, the cheese was removed from the pizza and processed alone. The resulting extracts were centrifuged twice for 30 min at 900× *g*, and the resulting supernatant was filtered through a Whatman #1 filter paper two times. Finally, the extracts were filtered stepwise, through a 0.8 µm filter followed by a 0.22 µm filter (MilliporeSigma, Burlington, MA, USA), aliquoted and stored at −20 °C until further use. Protein concentrations were determined by Bicinchoninic Acid Assay (BCA) using the Pierce^TM^ BCA protein assay kit (ThermoFisher, Waltham, MA, USA) according to manufacturer’s guidelines.

### 2.3. One- and Two-Dimensional Difference Gel Electrophoresis (DIGE) and Immunoblotting

The complete one-dimensional (1-D) and two-dimensional (2-D) difference gel electrophoresis (DIGE) analysis was performed by Applied Biomics (San Francisco) as described elsewhere [21]. The pool consisted of plasma from a subset of 10 allergic donors with sufficient plasma available. The pool was limited to 10 donors to avoid significant dilution of IgE to more rare milk targets that may only be recognized by one or two donors.

### 2.4. Image Scan, NanoLC-MS/MS and Data Analysis

Image scan and data analysis was performed as previously described [21].

### 2.5. Protein Band Quantification by ImageJ Analysis

ImageJ tool [22,23] was used on scanned western blot images. Distinct bands corresponding to molecular weights of known allergens found in the CM extract were selected for quantification analysis. Bands of interest were 14 kD, 18 kD, 22 kD, 30 kD, 55 kD, 67 kD, 80 kD and 160 kD. A profile plot for each lane was created based on the optical density of the extract bands. The area of the extract peak was selected and calculated.

### 2.6. PBMC Isolation and In Vitro Expansion of CM Extract-Specific T Cells

PBMCs were isolated by density gradient centrifugation from whole blood and cryopreserved, as described [24]. PBMCs were cultured with RPMI 1640 (Omega Scientific, Tarzana, CA, USA) supplemented with 5% human AB serum (Gemini Bio-Products, West Sacramento, CA, USA) at a density of 2 × 10^6^ cells per mL in 24-well plates (Corning, San Diego, CA, USA), in the presence of CM, custard, baked cheese or muffin extract (10 μg/mL). Cells were incubated at 37 °C in 5% CO_2_, IL-2 (10 U/mL; ThermoFisher, San Diego, CA, USA) and were added every three days after initial antigenic stimulation. After 2 weeks, cells were harvested, washed and screened for reactivity against the extracts by FluoroSPOT.

### 2.7. FluoroSpot

IL-5 and IFNγ production after CM and CM-containing food extract stimulation was measured by Fluorospot assay as described in another study [25]. In vitro expanded cells (1 × 10^5^ cells/well) were restimulated with the same extract as the original culture at 250 µg/mL, medium alone and PHA (10 µg/mL) as negative and positive controls, respectively.

### 2.8. Statistical Analysis

Analysis of Fluorospot data was using the following criteria, as previously published [21].

A minimum response of ≥100 SFCs per 10^6^ PBMCs, measured triplicates in response to a given extract were required to be significantly higher (*p* < 0.05) compared to background (assessed by Student T test, two-tailed, non-parametric), stimulation index ≥2, and finally, cytokine levels measured in response to medium alone (background levels) were subtracted from all data for each stimulus.

Statistical analysis for skin prick reactivity was performed using the Friedman test (paired non-parametric, two-tailed, corrected for multiple comparisons).

## 3. Results

**Figure 1:**
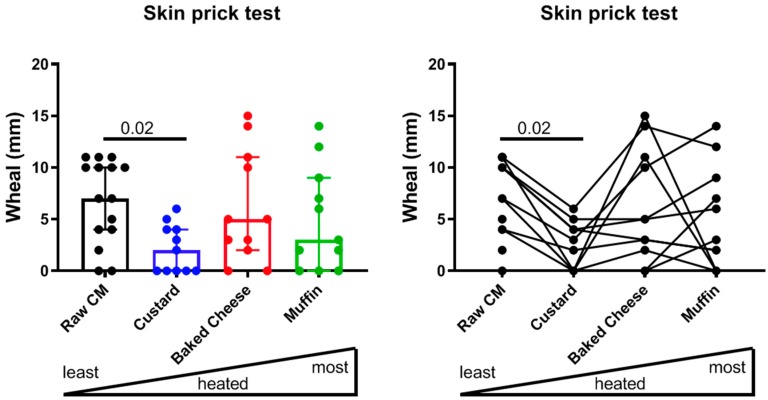
Skin prick test wheal diameters of CM-allergic subjects in response to CM (n = 15), custard (n = 11), baked cheese (n = 11) and muffin extracts (n = 11). The left panel shows a bar graph, with bars indicating median with interquartile range. The right panel shows a line graph with each individual represented by a connected line. Note, four donors tested for raw, pasteurized CM were not tested for the other three extracts. Statistical comparison was performed with the Friedman test (two-tailed), only considering the 11 subjects for whom full data sets were available. *p* < 0.05 is considered significant.

Information of allergy testing for all donors included in this study is summarized in Table 2. Strong correlations were observed for CM versus casein-specific IgE and IgG4 titers. In contrast, correlations between IgE titers and skin prick test results revealed no correlation for any of the extracts tested (Table 1). Further analysis of skin prick test data revealed that the largest SPT wheals were seen with the raw, pasteurized CM extract (median 7 mm), followed by extracts of baked cheese (median 5 mm), muffin (median 3 mm) and custard (median 2 mm) (Figure 1). SPT reactivity to custard extract was significantly lower compared to raw, pasteurized CM (*p* = 0.02). A modest trend for decreased median skin prick test reactivity was also observed for muffin extract compared to raw CM (Figure 1).

Using a plasma pool from 10 CM-allergic subjects, we performed an immunoblot analysis of the extracts to assess IgE reactivity on a protein-specific level. As a first step, we set out to determine the identity of dominant IgE-reactive protein bands using MS. The 1-and 2-D blots were inspected for bands that correspond in size to known milk allergens. Five prominent protein bands that run at molecular weights similar to known allergens were identified, cut out from corresponding SDS PAGE gels and analyzed by MS. For the selection of these bands, priority was given to bands prominent in the raw, pasteurized CM extract as this is the purest form of the allergen, and any selected proteins would not be traced back to elements of added food matrix. In addition to four bands selected from milk extract, an additional band from cheese was selected, as its size corresponded to Bos d 4 (~14 kD). Interestingly, there were several additional bands observed in the muffin extract. However, due to the abundance of non-milk proteins in muffin and the fact that these bands were not observed in any of the three other extracts, the additional bands in muffin extract were not further investigated.

**Figure 2:**
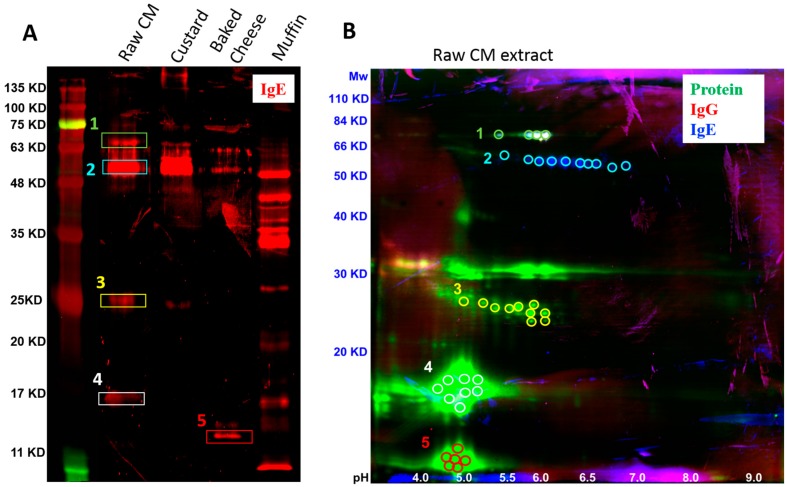
Immunoblot analysis of raw, pasteurized CM and CM-containing food extracts. (**A**) 1-D immunoblot analysis of raw, pasteurized CM, custard, baked cheese and muffin extracts showing IgE-reactive protein bands (red) and (**B**) a 2-D immunoblot analysis of raw, pasteurized CM extract showing protein spots (green) reactive with IgE (blue) and/or IgG (red) from a serum pool from 10 CM-allergic subjects.

Notably, four of those five bands were most prominently detected in the raw, pasteurized CM extract and the MS analysis was performed on the bands obtained from the raw, pasteurized CM extract. The fifth band, running at ~14 kD, was only detected in the baked cheese extract in the one-dimensional analysis. However, 2-D analysis of CM extract did reveal a very abundant protein at a similar size (Figure 2). Therefore, the MS analysis was done on the band originating from baked cheese extract for the 1-D blot but raw, pasteurized CM extract for the 2-D blot.

The highest confidence score for band 1 running at approximately 68 kD was serum albumin, also known as allergen Bos d 6 (Figure 2, Table 3), which is consistent with its reported molecular weight [10]. Band 2 running at 55–60 kD was also identified as serum albumin, suggesting that this allergen occurs in slightly degraded form, but is still IgE reactive. Both band 1 and band 2 were identified as serum albumin with high confidence in both the 1-D and 2-D immunoblots (Figure 2). The highest confidence score for band 3 (~25 kD) on the one-dimensional immunoblot was κ-casein (reported Mw 21.1 kD [26]) and serum albumin (Bos d 12 and Bos d 6, respectively). On the 2-D immunoblot, κ-casein was also identified as the top hit, however, the second highest confidence score was β-casein (29 kD [26]) (Figure 2, Table 3).

MS analysis of band 4 identified the protein as β–lactoglobulin (Bos d 5), for both the 1-D and 2-D immunoblots. This matches well with the reported molecular weight of β–lactoglobulin, 18.3 kD [27]. Finally, the smallest protein band running at ~14 kD was identified as κ-casein from the 1-D immunoblot of baked cheese extract (Figure 2A, Table 3). By contrast, in the 2-D immunoblot analysis of CM extract, the highest confidence score for the protein cluster at ~14 kD was α–lactalbumin (Figure 2B, Table 3). Interestingly, IgE reactivity was not detected for α–lactalbumin at 14 kD in the raw, pasteurized CM extract, in either the 1-D or 2-D immunoblots (Figure 2). The 14 kD size is consistent with the molecular weight for α–lactalbumin [27], whereas κ-casein has a molecular weight of 21.1 kD [26], suggesting that the band in the baked cheese extract may be a result of degradation.

**Figure 3:**
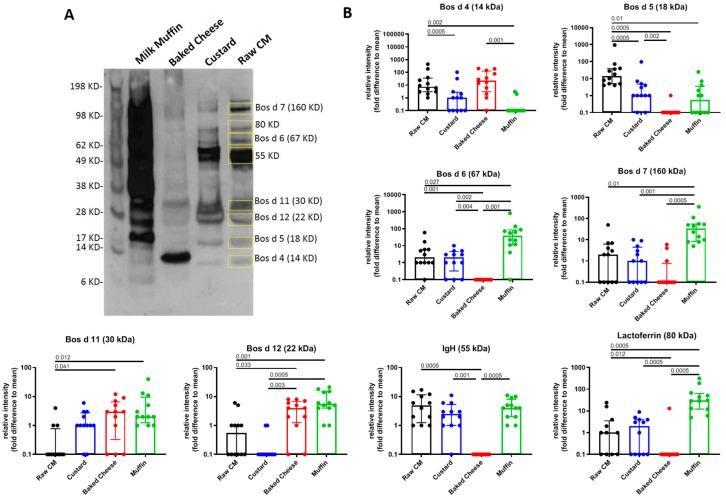
Quantification of IgE reactivity of allergen-corresponding protein bands in raw, pasteurized CM, custard, baked cheese and muffin extracts. (**A**) Representative western blot using an individual plasma sample, showing IgE-reactive protein bands. Bands that were quantified are shown in the raw, pasteurized CM extract lane, indicated by yellow boxes. Molecular weight markers (KD) are shown in the left side. (**B**) Bar graphs showing quantification of the eight protein bands (relative band intensities expressed as fold difference to mean) in the four extracts from western blots of individual subjects. Bars represent median, with error bars showing interquartile range, (n = 12).

In order to assess the effect of different levels of heating on CM allergens and their ability to bind IgE, we set out to quantify relative intensities of distinct protein bands corresponding to the major CM allergens or known proteins in the four different CM-containing food extracts. IgE-reactive protein bands were selected based on the raw, pasteurized CM extract (Figure 3A), and their relative band intensity was quantified using ImageJ software analysis. Overall, the level of retention of IgE reactivity across the different extracts was very dependent on the individual protein assessed. Notably, none of the proteins followed a pattern based on least to most heated. The most common observation was the abolishment of IgE reactivity amongst the proteins in the baked cheese extract, while the proteins retained some IgE reactivity in the other three extracts. This pattern was seen in five out of the eight proteins assessed: 18 kD (Bos d 5), 67 kD (Bos d 6), 160 kD (Bos d 7), 55 kD (corresponding in size to an immunoglobulin heavy chain- IgH) and 80 kD (corresponding in size to lactoferrin). While they all shared very low IgE reactivity in the baked cheese extract, the order of dominance in the remaining extracts still varied without correlating with degree of heating. Bos d 6 (serum albumin), Bos d 7 (immunoglobulin), and the 80 kD protein (lactoferrin) showed the most dominant IgE reactivity in muffin, followed by CM and then custard (Figure 3B). Bos d 5 (β–lactoglobulin) and the 55 kD protein (IgH) showed the most dominant IgE reactivity in raw, pasteurized CM followed by similar band intensities in custard and muffin (Figure 3B). The remaining three proteins all exhibited unique patterns. Bos d 4 (α–lactalbumin) was most dominant in baked cheese, followed by raw, pasteurized CM and custard. IgE reactivity to Bos d 4 in muffin was only detected in two out of 12 subjects (Figure 3B). Bos d 11 and 12 (β- and κ-casein, respectively) were both detected, most dominantly in baked cheese and muffin extracts, and exhibited surprisingly relatively low detection in raw, pasteurized CM extract. IgE reactivity to Bos d 11 was also readily detected in custard extract in contrast to Bos d 12, which was virtually undetected in this extract (Figure 3B). Although there was some variability among donors, the overall trends were consistent across the entire cohort. Reactivity trajectories for individual donors are depicted in the supplement Figure S1.

**Figure 4:**
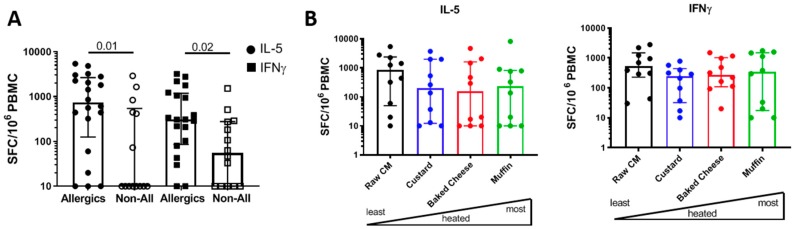
T cell cytokine production in response to raw, pasteurized CM and heated CM-containing food extracts. (**A**) A bar graph showing IL-5 and IFNγ production in response to re-stimulation with raw, pasteurized CM extract after a two-week expansion culture (allergic subjects n = 20, non-allergic subjects n = 14). (**B**) Bar graphs showing IL-5 (left panel) and IFNγ (right panel) production in response to re-stimulation with raw, pasteurized CM (black), custard (blue), baked cheese (red) and muffin (green) extracts after two-week expansion culture (n = 10). Bars represent medians, with error bars showing interquartile means. Statistical analysis was performed by Mann-Whitney test (two-tailed), *p* < 0.05 considered significant.

After evaluating the impact of different levels of heating on CM allergen-specific IgE binding, we set out to investigate if CM allergen-specific T cell reactivity is also affected. To confirm that T cell responses detected after in vitro culture with raw, pasteurized CM extract are antigen-specific and associated with allergic disease, we compared T cell responses from allergic and non-allergic children in response to raw, pasteurized CM extract side by side using IL-5 and IFNγ production as a read-out. As expected, CM-specific T cell reactivity was significantly higher in allergic compared to non-allergic subjects for both IL-5 (*p* = 0.01) and IFNγ (*p* = 0.02) (Figure 4A). Moreover, consistent with a Th-2 dominated allergic immune response, IL-5 production was higher in allergic subjects compared to IFNγ (non-significant trend), whereas in non-allergic subjects the opposite trend was observed.

Analysis of cytokine production in response to the different extracts revealed no significant difference in IL-5 and IFNγ production between the extracts (Figure 4B). A small decrease of cytokine production in response to custard, baked cheese and muffin as compared to raw, pasteurized CM is observed, and was slightly more pronounced for IL-5 as compared to IFNγ. Individual trajectories for T cell reactivity are shown in the supplement Figure S2.

## 4. Discussion

Years of clinical observation and some prospective studies have shown that CM-allergic patients often tolerate baked forms of CM before tolerating raw, pasteurized CM as they start to outgrow their CM allergy [7,9,28]. However, little is known about how allergic reactivity to cooked or baked CM-containing foods rather than raw, pasteurized CM is affected on an immunological level. In this report, we analyzed the IgE and T cell reactivity from CM-allergic patients in response to different extracts made from cooked or baked CM-containing foods versus raw, pasteurized CM extract. We observed significant correlations between CM and casein-specific IgE and IgG4 titers, however, comparison of IgE levels versus skin prick test results revealed no correlation. This lack of agreement between allergy testing methods has been reported previously [29]. Analysis of SPT reactivity revealed very slightly reduced wheal diameters in response to heated CM extracts compared to raw, pasteurized CM. Levels of reactivity were not directly related to the degree of heating of the respective extract, with custard extract eliciting the lowest responses, followed by muffin and then baked cheese. Milk in muffin and custard undergoes less food processing than milk contained in cheese. Therefore, it is important to highlight that immunological reactivity in response to cheese containing milk allergens or lack thereof may not only be related to simple heating, but is likely also altered by food processing involved in cheese production.

Based on the SPT data, we expected that the analysis of IgE reactivity would follow a similar trend, but surprisingly, we observed varied patterns. IgE reactivity was assessed by western blot analysis, quantifying the intensities of different protein bands corresponding to different allergens in each individual extract. Overall, IgE reactivity was not always lower in the heated CM-containing food extracts compared to raw, pasteurized CM extract, and no relationship with the degree of heating was observed. Several protein bands remained present and stable, sometimes even more intensely in the baked food extract compared to the raw, pasteurized CM extract. One major caveat of this analysis is the complexity of the extract and the identification of individual allergens solely based on their molecular weight according to where they are located in the western blot. To address this issue, we performed MS analysis of the four most dominant protein bands from the raw, pasteurized CM extract and one band from the baked cheese extract, which were not visible in the other extracts. For the CM extract, we also performed a 2-D immunoblot to add more resolution. These analyses revealed that although most protein bands were found to correspond to the expected allergen, degradation products and dimer formation are frequently observed and the identity of the protein band based on molecular weight alone is not sufficient for studies focused on individual allergens. Further, it is important to note that the food matrix in itself could impact IgE-binding. Our data indicate that in vitro IgE reactivity against cooked or baked CM-containing food extracts is not automatically reduced in proportion to the amount of heating the food has received. It is possible that molecular interactions between proteins or other components of the food matrix has a protective effect and prevents destruction of the IgE epitopes in the allergen. Moreover, due to sample limitations, assays were limited to IgE-binding studies. A functional assay such as the basophil activation test [30] may provide further insights into the impact of heating milk with respect to the allergic potential of the milk containing food.

Lastly, we investigated the effect of heating of CM on CM-specific T cell responses. Allergic T cell responses have been reported in CM allergy [31], yet it is unclear if heated forms of CM still retain their immunogenic potential to induce potent T cell responses. In line with previous reports on pollen-food syndrome [19], we observed no significant decrease in T cell reactivity against cooked or baked CM-containing food extracts compared to raw, pasteurized CM. The most likely explanation is that while heating can denature conformational epitopes and thereby reduce IgE binding, the short peptides recognized by T cells remain largely intact and are still processed and presented in the same form as they are in raw, pasteurized CM. In addition, it is important to mention that a food matrix may also have an impact on protein denaturation during heating. It has been reported that the presence of a food matrix delays the gastrointestinal digestion and reduces the absorption of allergens [32]. Assuming a similar principle, it is very possible that the food matrix from muffin, baked cheese or custard may impact the immunogenicity of some of the CM allergens.

The retained T cell reactivity is highly interesting in the context of clinical tolerance development. Studies have reported that desensitization during allergen-specific immunotherapy or even the natural development of tolerance is likely associated with a regulatory T cell response [33,34]. Therefore, it may be advantageous to retain T cell epitopes, as some of them may have the capacity to activate regulatory T cells and contribute towards tolerance induction. However, it is still not clear if regulatory responses target different epitopes than allergic T cell responses, or if it is simply a shift in the T cell phenotype. Moreover, the exact role of regulatory T cells and their contribution to tolerance induction in allergic disease is not fully understood.

Our study demonstrates that although heated CM-containing food extracts showed trends for decreased potency on in vivo testing and these foods are often tolerated clinically by CM-allergic patients, they can still elicit reactivity on an immunological level. While IgE-binding is somewhat impaired in the different extracts, it is not consistently reduced across all allergens. It has to be highlighted that in this study, we have only tested one batch per food extract. Variability in extract content has been reported for commercial extracts [25,35,36] and it is likely that reactivity reported herein will vary further when different extract batches are tested.

## 5. Conclusions

Overall, this study demonstrates that the process of heating CM contained in different food matrices alters IgE reactivity to some degree, but it is difficult to predict which allergens are affected and how. Further studies will have to be performed to provide details on how different CM allergens are affected individually in cooked or baked foods, and whether studying IgE or T cell reactivity to individual allergens after heating can shed light on the immune mechanisms involved and be informative in predicting disease prognosis.

## Figures and Tables

**Table 1:** Patient characteristics.

Variable	Milk Allergic (n = 20)	Milk Non-Allergic (n = 14)
Age in Years: Median (25–75%)	2.92 (1.25–5.69)	4.32 (2.44–10.75)
Male Sex (%)	13 (65%)	8 (57%)
Hispanic (%)	5 (25%)	1 (7%)
Race		
Black (%)	1 (5%)	0 (0%)
Caucasian (%)	7 (35%)	5 (36%)
Asian (%)	3 (15%)	4 (29%)
Other (%)	6 (30%)	5 (36%)
Multiple (%)	3 (15%)	0 (0%)
Additional Food Allergy (%)	19 (95%)	13 (93%)
Atopic Dermatitis (%)	18 (90%)	10 (71%)
Allergic Rhinitis and/or Environmental Sensitization (%)	16 (80%)	10 (71%)
Asthma (%)	8 (40%)	4 (29%)

**Table 2:** Baseline allergy testing and test correlations for cow’s milk allergic subjects.

Donor	CM- IgE (kU/L)	CM- IgG4 (mg/mL)	Casein- IgE (kU/L)	Casein-IgG4 (mg/mL)	CM Wheal Size (mm)	Muffin Wheal Size (mm)	Cheese Wheal Size (mm)	Custard Wheal Size (mm)
FA68P	10.7	n.d.	9.3	n.d.	n.d.	n.d.	n.d.	n.d.
FA92P	5.4	30.0	3.1	11.0	n.d.	n.d.	n.d.	n.d.
FA88P	1.2	11.9	0.0	0.2	10.0	4.0	5.0	6.0
FA84P	5.8	12.7	5.5	1.2	4.0	0.0	2.0	0.0
FA78P	9.5	7.3	9.6	0.0	7.0	3.0	10.0	14.0
FA85P	0.8	0.8	0.3	0.0	10.0	5.0	5.0	9.0
FA86P	10.9	6.7	0.0	0.0	0.0	0.0	0.0	3.0
FA97P	11.0	n.d.	2.0	n.d.	11.0	6.0	14.0	12.0
FA87P	19.5	9.8	19.4	1.3	n.d.	n.d.	n.d.	n.d.
FA90P	11.7	8.2	12.3	0.0	n.d.	n.d.	n.d.	n.d.
FA69P	10.4	n.d.	9.1	n.d.	11.0	0.0	0.0	7.0
FA76P	3.0	17.0	0.2	n.d.	11.0	n.d.	n.d.	n.d.
FA106P	10.6	6.3	2.4	2.7	0.0	0.0	11.0	0.0
FA110P	64.1	0.0	96.2	1.7	7.0	0.0	15.0	0.0
FA112P	1.3	n.d.	0.0	n.d.	10.0	n.d.	n.d.	n.d.
FA116P	0.0	n.d.	0.0	n.d.	2.0	n.d.	n.d.	n.d.
FA100P	5.3	n.d.	2.2	n.d.	n.d.	n.d.	n.d.	n.d.
FA114P	1.5	0.0	0.1	0.2	10.0	4.0	3.0	2.0
FA98P	0.0	n.d.	0.0	n.d.	5.0	n.d.	n.d.	n.d.
FA108P	25.4	n.d.	8.5	n.d.	4.0	2.0	3.0	2.0
Median	7.7	7.8	2.3	0.2	7.0	2.0	5.0	3.0
**Correlations**	**n.a.**	**n.a.**	**CM vs. Casein IgE**	**CM vs. Casein IgG4**	**CM IgE vs. CM SPT**	**CM IgE vs. Muffin SPT**	**CM IgE vs. Cheese SPT**	**CM IgE vs. Custard SPT**
R^2^	n.a.	n.a.	0.880	0.670	0.012	0.170	0.260	0.120
*p*-value	n.a.	n.a.	<0.0001	0.002	0.70	0.22	0.11	0.29

n.d.: not determined; n.a.: not applicable.

**Table 3:** Protein and allergen identification of IgE-reactive protein bands from western blot analysis of CM and CM-containing food extracts.

Band	1-D Blot (panel A)		2-D Blot (panel B)	
Size	Protein	Allergen	Protein	Allergen
**Band 1 (~65 kD)**	Serum albumin	Bos d 6	Serum albumin	Bos d 6
**Band 2 (~53 kD)**	Serum albumin	Bos d 6	Serum albumin	Bos d 6
**Band 3 (~25 kD)**	κ-casein, serum albumin	Bos d 12, Bos d 6	κ-casein, β-casein	Bos d 12, Bos d 11
**Band 4 (~17 kD)**	β-lactoglobulin	Bos d 5	β-lactoglobulin	Bos d 5
**Band 5 (~14 kD)**	κ-casein	Bos d 12	α-lactalbumin	Bos d 4

## Supplementary Materials

The following are available online at https://www.mdpi.com/2073-4409/8/7/667/s1, Figure S1: Line graphs showing IgE binding (relative intensity) to respective allergens in four extracts, raw CM, custard, baked cheese and muffin; Figure S2: Line graphs showing T cell cytokine production (spot-forming cells (SFC)) to four extracts; raw CM, custard, baked cheese and muffin.
