# Supplementary material for: Analysis of Allergen-Specific T Cell and IgE Reactivity to Different Preparations of Cow’s Milk-Containing Food Extracts
Source: Cells. 2019 Jul 2;8(7):667. doi: 10.3390/cells8070667 (PMC6679079; doi:10.3390/cells8070667)
Supplement: Supplementary file 1 [file cells-08-00667-s001.pdf]

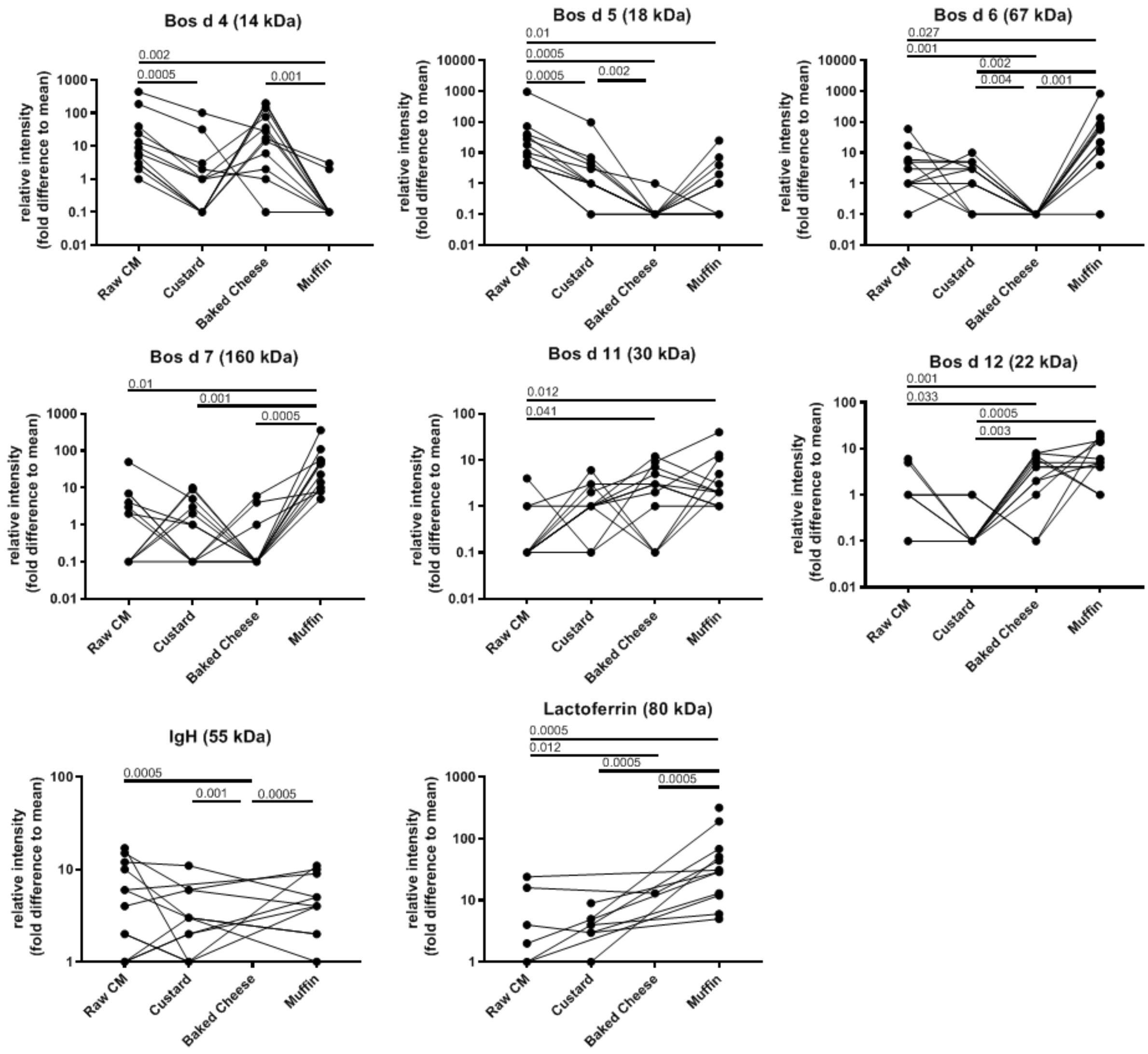

Figure S1. Line graphs showing IgE binding (relative intensity) to respective allergens in four extracts, raw CM, custard, baked cheese and muffin (n = 12).

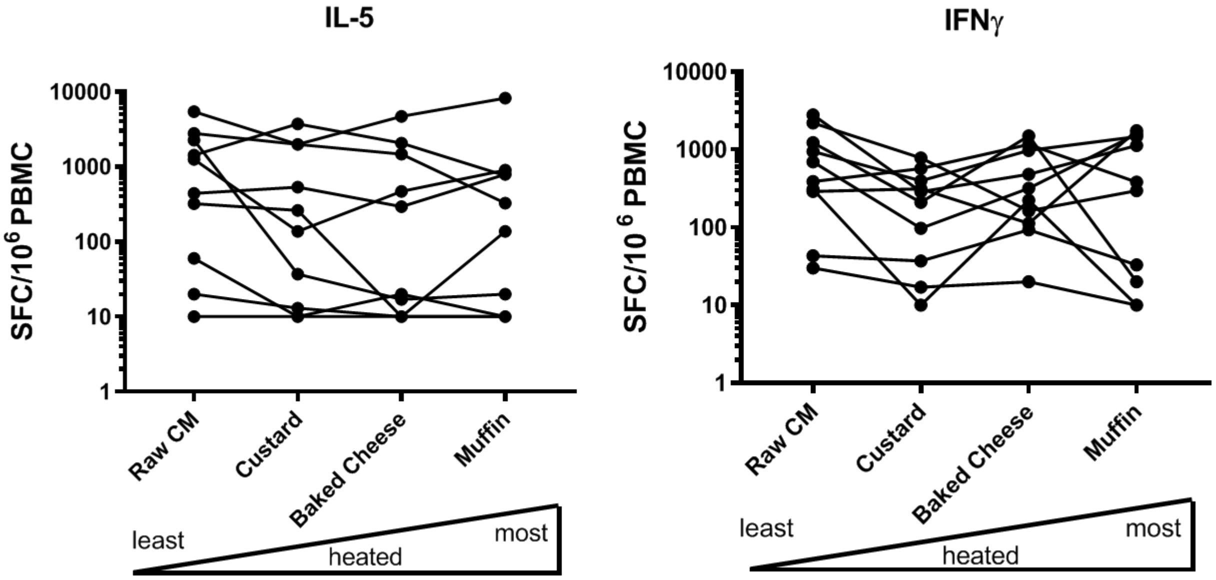

Figure S2. Line graphs showing T cell cytokine production (spot-forming cells (SFC)) to four extracts; raw CM, custard, baked cheese and muffin (n = 10).
